# Implementation model of data analytics as a tool for improving internal audit processes

**DOI:** 10.3389/fpsyg.2023.1140972

**Published:** 2023-02-10

**Authors:** Rubén Álvarez-Foronda, Carmen De-Pablos-Heredero, José-Luis Rodríguez-Sánchez

**Affiliations:** Departamento de Economía de la Empresa (ADO), Economía Aplicada II y Fundamentos del Análisis Económico, Facultad de Ciencias de la Economía y de la Empresa, Universidad Rey Juan Carlos, Madrid, Spain

**Keywords:** internal audit, internal audit function, data analytics, audit process automation, audit process improvement

## Abstract

**Introduction:**

The aim of this article is to understand the importance of internal audit departments todays—part of corporate governance and guardian of the organisation’s culture and climate—, as well as the opportunities that new technologies offer to increase their effectiveness and efficiency.

**Methods:**

To this end, based on an exhaustive review of the literature, the concepts of internal audit and data analytics are related, and a framework is proposed for the implementation of a technology of these characteristics in an internal audit department.

**Results:**

The results of the research show that those companies that invest resources in readapting their processes to technological change are likely to obtain better results than those organisations that keep their management procedures obsolete.

**Discussion:**

Based on these results, it is concluded that there is a need to consider technological change in internal audit departments, specifically data analytics, to increase the effectiveness and efficiency of audit processes.

## 1. Introduction

The modern origin of the internal audit function can be traced back to the financial scandals of the late 20th and early 21st century [the Enron, Tyco International or WorldCom scandals were particularly notorious (Drumea, 2008)]. History has shown that most major revolutions or changes in legislative oversight are preceded by an economic crisis or a financial scandal, and this was the case with the implementation of the Sarbanes-Oxley Act (SOX) in 2002 (Arjona-Canas et al., 2017).

The objective of the SOX Act encourages the participation and importance of auditors in the publication of transparent financial information (Fernández, 2007), which is why it is from this point onwards that internal audit departments took on special relevance.

The internal audit is an independent and objective assurance and consulting activity designed to add value and improve an organisation’s operations. In addition, it helps the organisation to meet its objectives by providing a systematic and disciplined approach to assess and improve the effectiveness of risk management, control and governance processes (IAIE, Instituto de Auditores Internos de España, 2017). It is also essential to highlight the role played by the function in achieving organisational objectives by being part of the company’s corporate governance, together with the audit committee, management, and external auditors (Gramling et al., 2004; Goodwin-Stewart and Kent, 2006). An internal audit, therefore, is a critical function in the task of control which helps to achieve corporate objectives.

Knowledge and understanding of the organisational culture play a key role in this task of defining and achieving objectives. On the one hand, organisational culture is the company’s identity card, aimed at influencing the deeds and actions of its stakeholders for the achievement of better business results (Panagiotis et al., 2014). In line with this idea, authors such as Gronewold and Donle (2011) argue that auditing the organisational climate and culture is an instrument that leads to a better understanding of stakeholder needs, engaging them at a strategic level, and leading to a better corporate image. In other words, organisational culture audits lead to better results.

On the other hand, information is a relevant resource to improve the decision-making process. In today’s ever-changing society, business decisions are made based on the information available (Rathi and Betala, 2019). Adequate knowledge and treatment of such information, which allows for more effective and efficient use of resources, is very important. A piece of data is a simple fact whose importance lies in its ability to combine to become information, which, when meaningfully valued, becomes knowledge for decision-making (IAIE, Instituto de Auditores Internos de España, 2019).

*Data analytics* is the science and art of discovering and analysing patterns, identifying anomalies and extracting other useful information in the underlying data (Byrnes et al., 2015). In other words, *data analytics* enables decision-making by establishing non-obvious relationships between data and transforming them into relevant and useful information for the organisation, information that would not be obtained through traditional data processing methods.

The audit sector is no stranger to this fact, where emerging technologies, most of which have information processing and management in common, have enabled the improvement of processes and, consequently, the evolution of organisations (López de-Sebastián-Miró et al., 2020). In the same vein, García and Navallas (2020) state that the large volumes of existing data have made new data analysis technologies essential in the field of auditing.

But aside from their undeniable benefits, one cannot ignore the fact that their use and implementation entail several associated risks, both economic and technological, as well as human (Islam and Stafford, 2021). As far as technological risks are concerned, it is important to highlight the security of the information involved in the processing and management of data. As noted by Broeders et al. (2017), expectations for *big data* are high but require additional safeguards to guarantee and protect citizens’ fundamental rights.

Regarding human risks, the 2030 International Standards for the Professional Practice of Internal Auditing states that internal audit resources should be appropriate, sufficient and effectively allocated to fulfil the department’s mission. The figure of the internal auditor requires an integrated and transversal knowledge of all areas of the organisation (De-Lara-Valero et al., 2017). To which must be included the need for training in the field of new technologies, such as *data analytics*, to ensure comprehensive training in the skills and knowledge necessary for the practice of internal auditing (López De-Sebastián-Miró et al., 2020).

As a result, the profile of the internal auditor has come to be considered as one of the best-qualified professionals within organisations (IAIE, Instituto de Auditores Internos de España, 2019), traits that should be taken into account when selecting this kind of profile by human resources departments. However, despite the literature available on the three research terms, internal audit, *data analytics* and human resources, few publications link the terms together, suggesting a lack of awareness of the important relationship these concepts have within organisations and for the future of the auditing profession.

Therefore, the main theoretical contributions of this study consist of covering the existing gap in the literature related to data analytics, human resources and internal audit, as well as exposing the main items to be taken into account when proposing to implement a technology of these characteristics. On the other hand, from a practical perspective, this work aims to provide a guide or model to serve as a reference for internal audit departments wishing to incorporate data analytics tools in their processes.

The present work has three main objectives: (1) To understand the relationship between the internal audit function, data analytics, and the management of the audit team; (2) To propose a theoretical model of methodology for the implementation of *data analytics* in an internal audit department, and (3) To analyse the relationship between the use of new technologies and the improvement of audit processes in terms of effectiveness and efficiency. In order to achieve these objectives, the work is structured as follows. The first section contains a literature review of all the factors that interfere with the audit process, as well as a descriptive analysis of the literature. The following section proposes a model for the implementation of *data analytics* in an internal audit department, along with a framework of best practices in the management of this process. Finally, the conclusions reached will be presented.

## 2. Methods

The method chosen begins with a review, analysis and discussion of the literature relevant to the study’s subject matter. A literature review is an account of what has been published, the purpose of which is to convey to the reader what knowledge and ideas have been established and what their strengths and weaknesses are, for further discussion. To carry out this literature review, the following steps have been considered: (1) Identify sources of information, (2) Identify and analyse the usefulness and relevance of selected publications, and (3) Bring together a number of independently conducted studies, sometimes with opposing results, and synthesise their findings (Rodríguez-Sánchez et al., 2019).

Three objectives are to be achieved through these phases: (1) To measure the evolution over time of publications on the subject studied, (2) To analyse the need to contribute complementary knowledge to the subject studied, and (3) To contribute to the dissemination of the importance, and increasing relevance, of internal audit departments in organisations.

The information-gathering process should avoid selection bias, hence the need to search as many sources as possible, and with appropriate selection criteria (Khan et al., 1996). For this purpose, the bibliographic search was carried out in three databases: *Web of Science* (hereinafter WoS), *Scopus* and the Institute of Internal Auditors of Spain (hereinafter IAIE). The first two have been selected because they are two of the world’s leading databases of bibliographic references and periodical citations, while the IAIE has been selected for its specialised nature in the subject matter, the development of the professional practice of internal auditing and, especially in recent years, its contribution to the disruptive potential of technology in the profession.

This analysis will be carried out in two phases. Firstly, the number of publications will be analysed according to previously defined search criteria, and the number of citations of the references obtained will be analysed (the IAIE database has been excluded from the relevance analysis due to its limitation in identifying its number of citations). Secondly, a study of the evolution of publications over time will be carried out to analyse the growing importance of the subject studied. To achieve the objectives laid out in this paper, a search for publications dated 19/05/2022 was carried out using the following search procedure (Table 1).

**Table 1:** Search procedure.

Databases	*WoS*	*Scopus*
Scope	Worldwide scientific production	Worldwide scientific production
Characteristics	Quality indicators: JCR, no. of citations, quartiles.	Quality indicators: SJR, no. of citations, quartiles.
Inclusion criteria	*Topic*	*Topic*
Time range	Every year until 2022	Every year until 2022
Search date	May 12, 2022	May 12, 2022
Search terms	“Internal Audit*”	“Internal Audit*”
	“*Data Analytics*”	“*Data Analytics*”
Initial results	23	28
Inclusion criteria	Articles	Articles
	Book chapters	Book chapters
	Review	Review
Results	18	23
Filtering by áreas	*“Management”*	“*Business, Management and Accounting”*
	*“Business Finance”*	*“Economics, Econometrics and Finance*”
Results	12	17
Filtering	Duplicates	Duplicates
	Authors not identified	Authors not identified
	Not related to the *topic*	Not related to the *topic*
Final results	11	15

Own elaboration.

**Figure 1:**
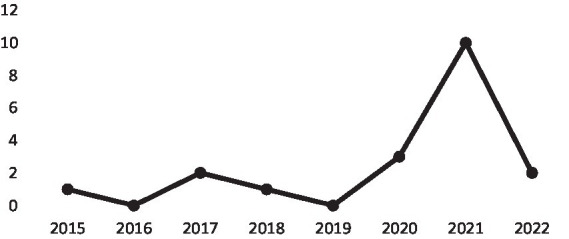
Time evolution of publications. Own elaboration)

It is important to establish a temporal progression in the publication of articles that allows us to analyse the increasing relevance of the study we are preparing to carry out. As can be seen in Figure 1, interest in the research topic is increasing over time, with an above-normal trend from 2020 onwards. This increase can be explained if we look at global economic and cultural developments in that period.

As discussed above, the SOX Act (2002) can be considered the modern origin of the internal audit function. However, this would not explain the considerable upturn in the publication of articles from 2020 onwards. This is because SOX addresses financial transparency issues but does not link the internal audit function to new information technologies.

A convincing explanation for this can be found in the shift in the profession’s approach, an evolution that views emerging technologies as audit tools for the present and the future (López De-Sebastián-Miró et al., 2020).

It is therefore the emergence of new technologies, specifically *data analytics*, that has led to a surge in publications analysing the relationship between these two concepts, internal auditing and *data analytics*. In this regard, it is worth noting that thanks to the expansion and adoption of *data analytics*, many organisations have focused on converting big data and incorporating it into their technology infrastructure (Deloitte, 2016).

## 3. Analysis model: Success factors in the implementation of data analytics in internal audit departments

Businesses need revenue to survive, just as they need their core business to function in order to be profitable. However, in setting and achieving objectives, we must not forget a concept that, from now on, will become particularly important: risk. The organisation must consider the risk it will take to achieve its goals, namely its risk tolerance, both in its quantitative and qualitative variables (IAIE, Instituto de Auditores Internos de España, 2017). In this regard, Nordal et al. (2017) highlights the impact of organisational culture on the identification and treatment of risks.

This is where the internal audit function, through its assurance, risk management and corporate governance activities (Rivero, 2016), plays a leading role, providing an added value that is both necessary and fundamental for the organisation’s survival. The internal audit function, therefore, helps to improve the organisation’s control environment, as well as the management and administration of risks following the analysis and evaluation of the different business processes (Arjona-Canas et al., 2017).

However, the importance of the internal audit function as a driver of change and a generator of added value in organisations (Arjona-Canas et al., 2017) has progressed from a traditional approach to a modern one, largely driven by the advent of new data analytics technologies, providing a more effective assurance with a more efficient use of resources. *Data analytics* can thereby improve the performance of the internal audit function (Smidt et al., 2021).

Although it is true that at the beginning of the 20th century the internal audit function had a generally financial perspective (Villegas, 1992), this study aims to analyse its more operational approach. Internal auditing is seen as an independent activity of supervising compliance with rules, policies and procedures through internal control, evaluating its design and effectiveness, and becoming an advisory function for the company and its owners (López et al., 2003).

However, to achieve this improved process through the implementation of *data analytics* technology, a number of factors must be considered that will determine the success of the change (Islam and Stafford, 2021). Similarly, any process re-engineering through technology has to be approached according to certain phases (Hammer and Champy, 1994).

To this end, IAIE, Instituto de Auditores Internos de España (2019) proposes that problem-solving should be approached from six distinct phases: The identification and definition of the problem, the search for and development of alternatives, the evaluation and comparison of alternatives, the selection, the implementation of said selection, and the monitoring and follow-up.

In this study, the incorporation of *data analytics* for the improvement of internal audit processes, it is deemed appropriate to simplify the six phases proposed above into four. The purpose of this simplification is not to eliminate any of the phases suggested by the IAIE, but rather to group some of them as they are indirectly included in the four phases proposed for this study, to simplify and focus the reader’s attention. Thus, the phases considered are as follows: (1) Identification of what is needed, (2) Cost–benefit analysis, (3) Decision on technological implementation, and (4) Monitoring and follow-up of results.

### 3.1. Identification of needs

The International Standards for the Professional Practice of Internal Auditing states in its Standard 2000 (IAIE, Instituto de Auditores Internos de España, 2017, p. 43) that the internal audit activity adds value to the organisation and its stakeholders when it considers its strategies, objectives, and risks, delivers process improvements, and objectively provides assurance.

To provide this assurance, the 2010 Standard (IAIE, Instituto de Auditores Internos de España, 2017, p. 43) indicates that the director of internal audit (hereafter, DEA) should establish a risk-based audit plan consistent with the organisation’s goals, with its corporate culture. This indicates that the internal audit function adds value when it provides risk assurance in accordance with the requirements and needs of its stakeholders, both internal and external to the organisation.

Argue for the strong relationship between the strategic level and the other levels of the organisation as a key factor for joint success. Other authors such as Andrade et al. (2021) state that the choice of audit projects must be made meticulously, so that they are aligned with the strategy of the organisation to which they belong.

Therefore, audit processes should be focused on the key risks that threaten the organisation’s operational objectives. This is known as a risk-based internal audit (Coetzee and Lubbe, 2013).

While it may seem that auditing processes based on their risk are widely accepted, studies such as the one conducted by Coetzee and Lubbe (2013) show that while private companies enjoy a high level of risk maturity, this is not the case in public sector organisations. Thus, it is clear that the consideration of risk in the organisation has not traditionally been seen as it is today and depends to a large extent on the company’s climate and culture.

In this sense, Reichers and Schneider (1990) state that the organisational error climate is part of its culture and comprises beliefs that are considered commonplace concerning error handling in an organisation. As such, in a climate of high error management, it is recognised that errors are likely to occur every time people perform different tasks (Gronewold and Donle, 2011), so managing the organisational climate is seen as a key determinant of the internal audit function.

In addition to the assurance function, an effective internal audit helps the organisation to improve its internal controls. The internal auditor should participate in corporate governance by assessing the internal controls in different areas of the organisation (Singh, 2003), and proposing improvements. This way, the internal audit department contributes to the organisation’s governance (Hu et al., 2021) by assessing its internal controls.

Therefore, it can be said that the internal audit function has a dual objective, on the one hand, to provide assurance, and on the other hand to improve governance. Both functions should be considered when drawing up an audit plan based on existing risks within the organisation.

Using the risk factor as the basis on which the internal audit plan is prepared will allow prioritisation of the higher-risk areas of the company over the lower-risk areas. This approach ensures that the risks are within acceptable and tolerable levels for the organisation (Coetzee and Lubbe, 2013).

However, risk-based planning requires prior knowledge of risk. It is important to note that an organisation’s risk map does not remain static but is an ever-changing variable over time and therefore needs to be updated with sufficient frequency to always provide a true picture of the organisation’s situation.

In this regard, the Institute of Internal Auditors (IIA) establishes in its standards of good practice that the level of risk should be identified and assessed at least once a year (Institute of Internal Auditors (IIA), 2022, standard 2010.A1). Traditionally, these risk measurements have been performed based on results of audits already carried out and historical information. This poses a constraint, since, if the current situation of the company is to be known, it will be calculated based on the results from previous months, and even years.

Silva and Almeida (2014) reinforce this limitation by suggesting that the traditional approach to internal audit project management is based on consulting data, facts and records of past projects, which may be insufficient for the current scenario, marked by greater access to information and technologies.

However, *data analytics* provides an alternative to this limitation. Compared to audit plans based on historical information, *data analytics* offers the possibility of knowing the current risk situation of each of the areas of the organisation at any given moment in time. This applicability of data analytics is supported by studies such as the one carried out by Broeders et al. (2017), who state that, although big data can be used to carry out historical analysis, its true potential lies in its predictive and real-time use, which provides the ability to predict the future with a high degree of probability.

Furthermore, in this process of incorporating technology into the internal audit function, it is essential to have the organisation’s support. The perceived need for adopting technology in audit processes will depend on the organisation’s perception of its ease of use and usefulness (Kim et al., 2009), as well as the existing conditions that facilitate it (Mahzan and Lymer, 2014).

Furthermore, it is considered relevant to include an additional element to the ease of use and usefulness, the real need to introduce a change in a process that is already being carried out with other simpler and less expensive tools such as Excel. Again, *data analytics* provides an answer to this question by providing an increase in the processes’ effectiveness and efficiency (Smidt et al., 2021), which we do not obtain with other tools. In this sense, only those companies that adopt a change in their processes will be able to maintain and increase their competitive level in the future (PwC, PricewaterhouseCoopers, 2012, p. 2).

However, the will to change is of no use if there is not adequate involvement of the company’s owners, managers and human resources. In this sense, and as the internal audit is an independent assurance unit (IAIE, Instituto de Auditores Internos de España, 2019), it is essential to have the support and direct involvement of the company’s management, who must provide sufficient confidence to carry out the necessary changes to adapt to the market’s most current and sophisticated technologies. In other words, a change of mentality is needed that considers the internal audit department as a driver of change in the organisation (Arjona-Canas et al., 2017).

Future success will depend on this ability to align the technological interests of the department with the interests of the different stakeholders, since only with a good organisational culture and a desire for technological change can the desired goals be achieved (López De-Sebastián-Miró et al., 2020).

Therefore, it can be deduced from the above that the improvement of audit processes, understood as an increase in their efficiency and effectiveness, as well as real-time knowledge of the organisation’s risk, are the main triggers for the process of implementing *data analytics* in an internal audit department.

### 3.2. Cost–benefit analysis

Once the current needs of the organisation have been satisfactorily defined and analysed, an updated view of the company’s risk has been obtained and the department’s objectives have been aligned with the corporate objectives, a technological acquisition project will be proposed. For this task, the second phase of the change’s implementation will consist of an evaluation of the expected cost–benefit:

Cost–benefit analysis is defined as a systematic approach designed to analyse the strengths and weaknesses of different alternatives, through an investment plan according to the expected benefits to decide which one best meets the organisation’s interests (David et al., 2013).

Data analytics brings a number of indisputable benefits, but whether the expected results justify the costs associated with the investment must be analysed (Ngulube, 2011).

Additionally, obtaining the necessary funding for the investment will be more difficult if this prior analysis is not available (Aguilera-Díaz, 2017).

When carrying out a project to implement technology such as data analytics in an internal audit department, a series of factors or facilitators must be taken into account, and the success or failure of the project will depend on their correct management.

Table 2 sets out the main items to be taken into account when carrying out the cost–benefit analysis for the implementation of technological change, differentiating them according to the phases of the standard audit in which they occur.

**Table 2:** Cost–benefit analysis of data analytics implementation.

Standard audit phases	Costs	Benefits
Planning	Software Acquisition Training Investment in human factor	Updated risk status of the audited unit
		Trend analysis
		Previous population analysis
		Sampling optimization
		Resource allocation optimization
Execution		Fraud detection
		Resource optimization / Automation
		Audit risk reduction.
Reporting		Visibility
		Impact
		Reliability
Follow-up of recommendations		Continuous Auditing
		Cost reduction

Own elaboration.

The project of implementing data analytics in an internal audit department suggests that it be analysed considering the 4 fundamental phases into which a standard audit work is divided, phases established on the basis of the guidelines and recommendations issued by the IAIE: planning, execution, reporting and periodic follow-up (IAIE, Instituto de Auditores Internos de España, 2019).

Before analysing each of the four phases described above in detail, Table 3 considers providing a rough estimate of the time, calculated as a percentage of the total time spent on a standard audit and in each phase. The objective is to establish a starting point for understanding the phases of the process to which more resources and effort (higher risk) should be devoted, as well as to establish the basis for interpreting the project’s final result and objective, reducing and transferring costs from one phase to another.

**Table 3:** Estimated percentage of time dedicated to each phase of a standard audit.

Phase	% of time over total audit time
Planning	20%
Execution	65%
Reporting	15%

Adapted from the phases proposed by the Institute of Internal Auditors of Spain, 2019.

In this case, the follow-up phase has not been considered in this estimate as it is not directly related to the audit, but is linked to it as a subsequent and complementary phase.

Computer-assisted auditing techniques will be used by auditors to improve audit efficiency, uncover operational problems, and achieve more effective monitoring (Fan, 2020). Therefore, once the point of origin of a standard audit has been established, the four phases described above are detailed to analyse the possible improvement of processes to conclude what percentage of time can be saved in each of them, time that can be regarded in two different ways: effectiveness and efficiency.

Efficiency: This is understood in two ways. On the one hand, a reduction in audit costs through a reduction in the time spent on operational and routine tasks—Optimisation of the audit—(Rojas-Amado and Escobar-Ávila, 2021). On the other hand, cost transfer: through an analysis of the dedication percentage in each of the phases, a reallocation of resources can be carried out, so that we manage to increase the overall efficiency of the audit by dedicating the greatest volume of resources to the phases that require it most.

Effectiveness: using data analytics allows for the analysis of the total population and of information that could hardly be carried out using manual techniques (IAIE, Instituto de Auditores Internos de España, 2019; Oliván-Mainar and Fernández-Vicente, 2021).

#### 3.2.1. Phase 1: Planning phase

In today’s business context, where all organisations generate large volumes of data continuously, it is particularly relevant to have a tool for processing this data (Islam and Stafford, 2021). Traditionally, this task has been carried out manually and tediously. However, with today’s tools, this work of acquiring and processing data to obtain useful knowledge becomes much more affordable, effective and efficient.

Due to the globalisation and digitalisation of companies, risks are changing faster and faster due to the dynamic risk environment, so a flexible audit plan is needed to cover the uncertainty and complexity in companies (Eulerich et al., 2020).

Planning occurs before conducting fieldwork or audit testing, the risk assurance. In this stage, the objectives and scope are established. It begins with the definition of the scope and ends just before the execution of the tests. For the purpose of this study, audit planning will be considered in the two aspects in which *data analytics* technology can be incorporated: preparation of the overall audit plan, and planning of specific audit tasks. In other words, planning aims to objective is to create an audit plan that is carried out effectively [Normas Internacionales de Auditoría, (NIAs). n.d., 300.4].

In this phase, the expected benefits relate to the information hidden within the data; the aim being to uncover non-obvious relationships between said data, pearls of information that usually go unnoticed (IAIE, Instituto de Auditores Internos de España, 2019).

In the definition phase of the annual audit plan, as well as the planning of specific tasks, studies such as “elevating internal audit” carried out by PMP (2021), suggest that the main benefit of *data analytics* will be to use this hidden information to guide the allocation of resources or, in other words, to use this technology to understand the company’s situation, identify trends and carry out population analyses that allow us to better focus the audit work based on the risk they present. As such, investments in technology have been shown to improve the breadth and depth of audit coverage (Protiviti, 2021).

As an example, if a model audit of the procure-to-pay process is analysed, the use of *data analytics* will allow to know, prior to any testing, the status of all transactions that have taken place in a given period of time. From this prior knowledge, information on the weaknesses and strengths of the departments involved in the process will be extracted in order to plan the audit based on the actual situation at a given point in time. The aim is therefore to focus attention and efforts on those phases of the procure-to-pay process that require it most.

This finding is also shared by West et al. (2005), who stated that even a fraction of an improvement in prediction quality can result in financial savings (Hu et al., 2021).

#### 3.2.2. Phase 2: Execution phase

Audit testing is the process by which risk assurance work is carried out (IAIE, Instituto de Auditores Internos de España, 2019). All units or departments of an organisation carry out a series of activities that are associated with risks inherent to the process. Thus, in order to control or reduce such risks to a tolerable level, a series of controls are established to mitigate these risks. The execution phase consists of testing that these controls are working correctly, thus reducing the risk to a residual and manageable level for the organisation (IAIE, Instituto de Auditores Internos de España, 2019).

The audit team will perform tests, collect evidence, analyse data and information, and draw conclusions and recommendations. The IAIE, in its Manual “Internal Audit Practice” (2019), states that the execution of audit tests can be considered one of the critical phases of the audit process, as it is at this stage that evidence is gathered, data is evaluated, and observations and recommendations are made, all of which require a large number of resources. This makes it the key phase for the implementation of *data analytics* technologies (Rojas-Amado and Escobar-Ávila, 2021).

The key benefit of *data analytics* in this phase will be its ability to automate testing. This will reduce the time spent on testing. Furthermore, the analysis of non-obvious relationships between data provides other benefits that would not be achieved by manual analysis, such as fraud detection and reduced audit risk (Schneider and Dai, 2015). The latter position is reinforced by studies such as the one conducted by Islam and Stafford (2021), who identified that in highly regulated industries, where one of the audit objectives is fraud detection, the likelihood of data analytics adoption increases by 8.5%. It is also important to note that the study focused on the adoption of *data analytics* in five key areas of the company: population testing, process improvement, compliance, risk control and fraud management. As can be seen, these areas overlap with those mentioned in this study.

However, the use of massive information at this stage is not without risk. Too much information can confuse users, as well as lead to biased judgements (Hu et al., 2021). To address this issue, *data analytics* aims to help users identify and analyse information more quickly and provide more reliable results (Hu et al., 2021).

Moreover, with regard to the reduction of audit risk, it is worth mentioning that the use of advanced audit tools allows for a population-based analysis of all transactions, replacing the sample-based study that has been carried out until now. Authors such as Feung and Thiruchelvam (2020) point out that *data analytics* represents a paradigm shift from a traditional risk-based sampling and auditing approach to a whole population verification approach.

The importance of replacing the sample analysis becomes evident if we take into account that a non-conformity, or deficiency not detected in a sample selected by the auditor, could extend its effects, causing inaccuracies in the rest of the processes (Andrade et al., 2021).

Therefore, the benefits of the execution phase can be synthesised in the reduction of audit risk (greater effectiveness), as well as the automation of tests and reduction of time spent (greater efficiency; Oliván-Mainar and Fernández-Vicente, 2021).

#### 3.2.3. Phase 3: Reporting of results

One of the fundamental reasons for the existence of the internal audit function is the concern of stakeholders for the proper functioning of the organisation. In this sense, authors such as López De-Sebastián-Miró et al. (2020) point out the importance of providing the Audit Committee and senior management with relevant information for decision-making.

The reporting phase consists of communicating audit results to stakeholders (IAIE, Instituto de Auditores Internos de España, 2019). This communication is as important as the execution work, as poorly worded or poorly reported results detract from the added value of the work performed. The audit report is also critical because it acts as a catalyst for change (IAIE, Instituto de Auditores Internos de España, 2019). This highlights the exciting possibilities of *data analytics* in this phase, as it offers greater visibility and impact in the communication of audit findings.

On the other hand, in multinational companies, where the internal audit function is centralised in a single location (usually the Group’s parent company), having a tool for the continuous exchange and analysis of information becomes a huge advantage, as it allows us to know the real situation of any of the subsidiaries at any given time without the need to carry out a complete audit.

The primary objective pursued with *data analytics* in this phase is, therefore, to report the identified audit findings in a more visual and useful way.

#### 3.2.4. Phase 4: Follow-up phase

In addition to the previous phases, it is necessary to include an additional one due to the importance that is drawn from it, the follow-up of recommendations (IAIE, Instituto de Auditores Internos de España, 2019).

The IAIE, Instituto de Auditores Internos de España (2019) states that the follow up of recommendations consists of providing assurance to stakeholders that deficiencies identified during audits have been corrected or, while not 100% corrected, have mitigated the risk to a level that is acceptable to the organisation.

The application of *data analytics* in this process will consist of using the automated testing spectrum, scheduling it over time thanks to applications embedded in the analytics tool, and automating the entire follow-up process, from testing to the reporting of findings. In this sense, Andrade et al. (2021) point to *data analytics* as the leading tool used to carry out a continuous audit of processes.

Finally, it is essential to highlight an element common to all the proposed phases. Thus, studies have shown that the use of *data analytics* in auditing represented a 44% reduction in time spent in the execution of audits compared to the same scenario prior to the implementation of the tool. Considering the number of tests performed, there was an increase of 80%, not to mention the increase in the sample size, which now considers 100% of the population of the audited period (Andrade et al., 2021).

### 3.3. Technology implementation decision

Once the two previous phases (identification of needs and cost–benefit analysis) have been successful, the decision to implement *data analytics* in the internal audit department will be made. However, despite the success of the two previous phases, the project’s success factors must be considered. Thus, Table 4 sets out the main factors to be taken into account before making the final implementation decision.

**Table 4:** Factors to consider in the successful implementation of data analytics.

Standard audit phases	Factors
Planning	Technological situation of the organisation
	Degree of digitalization
	Innovative culture / Willingness to change
	Existence of adequate and sufficient resources
Execution	Staff qualification / Trainning
	Flexibility
	Medium and long term mentality
	Resistance to change
Reporting	Awareness of the management layer
	Resistance to change
	Medium and long term mentality
Follow-up of recommendations	Medium and long term mentality
	Collective involvement of other areas

Adapted from the phases proposed by the Institute of Internal Auditors of Spain, 2019.

### 3.4. Monitoring and follow-up of results

“No problem-solving process is complete until the impact of the selected alternative has been monitored and evaluated and, if the problem that motivated the change still exists, the resolution cycle will need to be started again” (IAIE, Instituto de Auditores Internos de España, 2019).

Thus, based on the definition provided, monitoring and follow-up is the quantitative and qualitative evaluation of the decision taken, comparing expectations and results, and analysing whether the technological change incorporated has been satisfactory.

In addition to the factors detailed in each of the previous phases, other facilitators must be considered when preparing to carry out a project of these characteristics. Because of their global scope, they are not unique to the internal audit department but affect the organisation as a whole. These factors or enablers are as follows:

#### 3.4.1. Degree of digitalisation

The degree of digitisation in the company is a prerequisite for carrying out this type of project. *Data analytics* tools aim to access data, process it, and draw conclusions. The ability to identify and evaluate unstructured data will lead to better audit evidence and facilitate the effectiveness of internal control (Schneider and Dai, 2015). Data processing or data mining is the process by which data are grouped and classified according to their characteristics (Yang et al., 2022).

However, this integration process of *data analytics* would not be possible without adequate access to data, i.e., without the data being organised and digitised in the organisation’s IT systems. Digitalisation is the process of introducing digital technologies, which essentially deal with changes caused by information technologies (Nathanael and Sarens, 2021).

Therefore, the inclusion of advanced auditing technologies is most prevalent in large organisations with advanced information management systems, such as Enterprise Resource Planning (ERP) systems, where information, data, and access to data is stored and made easily accessible.

However, the availability of information in complex integrated systems cannot be without risks. The link between the company’s information system and business strategy has a certain effect on the achievement of the organisation’s goals (Hu et al., 2021). Authors such as Morales et al. (2022) point out that in a context in which the internal auditor is dependent on the information contained in a computer system, it is essential to carry out an analysis of the integrity of the information contained therein to guarantee its reliability.

In conclusion, the larger the organisation, the greater the need for advanced information management and administration systems.

#### 3.4.2. The human factor

The human factor is the other major enabler that will be considered indispensable to our study. As with digitalisation, this factor is common to all phases of the audit process. In turn, the human factor analysis will include the degree of involvement, training, and the level of acceptance and management of change.

In recent years, the internal auditor profile has become one of the best-qualified professionals within the organisation (IAIE, Instituto de Auditores Internos de España, 2019). When using *data analytics*, the auditors’ IT skills are key to driving its adoption (Krieger et al., 2021). This new IT competence, far removed from the more traditional financial profile (Villegas, 1992), poses an added difficulty in the search for internal auditor profiles. Therefore, to achieve process improvement based on *data analytics*, IT knowledge and critical thinking skills are indispensable (Islam and Stafford, 2021).

In this sense, it is evident that it is difficult to adapt to technological change when it is observed that Microsoft Excel is still the software tool most used by internal audit departments (Smidt et al., 2021).

However, to overcome this training obstacle, a multitude of data analysis tools have now been developed that incorporate a relatively simple user-machine interaction interface, so that anyone, once trained in the use of the tool, can programme execution scripts without the need for extensive programming knowledge.

Lastly, an in-depth examination of change management and the audit team’s resistance to change is considered relevant. Authors such as point to the importance of the acceptance of change by the audit team as part of the success of the change process. In this regard, other authors such as Dai (2017) add the importance of there being an organisational culture of support for change from the executive layer of the organisation.

Introducing a new technology entails an extra effort on behalf of all stakeholders (training, investment in time, etc.). However, within this human resources management, special attention must be paid to how change is managed within the department, where overcoming barriers such as resistance to change or attachment to traditional operations can jeopardise the viability of this form of innovation project.

During the integration process, efforts should be made to reduce the complexity of the transition by clarifying the status and roles of each person, thereby simplifying the process (Rodríguez-Sánchez et al., 2017).

## 4. Discussion and conclusion

In the last century, there has been a series of financial scandals involving large corporations which, in many cases, have cast doubt on the good practices carried out by such organisations. This type of malpractice has a direct impact not only on the companies themselves but also on society as a whole, whose interests are often linked to their good practices.

In this task of protecting the interests of all parties involved, the figure of the internal auditor plays a fundamental role. The role of the internal auditor, as a means of guaranteeing the transparency and good practice of companies, becomes the main mechanism for defending the organisation’s interests, ensuring its continued existence, and limiting the intentional malpractice exercised by a minority of people concerned only with their own personal interests.

In this risk assurance and control function, internal audit departments must be aware that strengthening control through technology can reduce, and even eliminate, the impact of human error.

In carrying out this task of improving organisational control, *data analytics* can provide an extraordinary advantage. Through this type of tool, not only is progress made in detecting fraud but it also allows audit processes to evolve, increasing their effectiveness and efficiency. Wang and Cuthbertson (2015) note that *data analytics* allows auditors to perform predictive analytics to examine and identify patterns in data that do not conform to expected patterns. Similarly, this type of tool has allowed for the automation of tests and the identification of risks almost in real time. It has led to a change in the paradigm of a profession that is increasingly oriented towards a continuous auditing model, as opposed to the traditional conception of static auditing.

Therefore, the use of data analytics as an audit tool makes it possible both to reduce work times and restructure the time invested in each of the audit phases (efficiency), such as extending the audits’ scope, extrapolating the reviews to the entire population as opposed to traditional sampling (effectiveness).

In this way, and in response to one of the three objectives of this study set out in the introduction, it is concluded that the use of auditing techniques using new information technologies increases the effectiveness and efficiency of auditing processes.

However, apart from the undoubted benefits that *data analytics* offers, there are certain risks when implementing this technology. Risks such as information security, resistance to change, the associated cost versus the expected benefit, or the handling of enormous amounts of information, have led to the need to establish an implementation model that combines technology and people, which serves as a reference framework when attempting to carry out a project of these characteristics.

The results of our study suggest that such a project cannot be undertaken without considering a number of factors associated with each stage of the auditing process. Apart from the expected costs and benefits, the degree of a company’s digitalisation, as well as the human factor, are key elements whose correct management will determine the success or failure of the innovation project.

In this regard, it is important to stress that this technology is not intended to supplant the role of the auditor, but rather to complement and, above all, improve it. As the great technological changes throughout history have shown, evolution does not destroy jobs, but rather transforms and reinvents them in a way that results in progress in its more traditional approach. Thus, it can be argued that the internal auditor’s role is enhanced by *data analytics*, while data analytics builds on the auditor’s knowledge.

This combination of human and technological factors responds to another of the objectives set out in our study. The conception that a project of these characteristics implies a change in the mentality of the auditors, and the fact of considering human resources management as a key factor will mark the success or failure of the project.

Finally, the use of technology as a process improvement tool is not new to the business sector. *Data analytics* tools are already in use today. However, their use is not yet widespread in internal audit departments and there is a great lack of knowledge of both the benefits that can be obtained and how to incorporate technology into their internal processes. Through this research, an implementation guide or model has been developed that allows companies to have a reference on how to carry out a project of these characteristics. Therefore, with the definition of this implementation model, the third of the objectives set out in this work has been achieved.

In short, this new scenario, which is much more technological than the previous one, requires internal audit departments as a whole to bring together new skills and tools that allow for proper risk control in the organisation, an anticipation of future events, and a continuous updating to current trends. In other words, internal audit departments need *data analytics* technology to improve their processes.

### 4.1. Limitations of the study

The results of this research should be interpreted with caution due to the inherent limitations of the methodological process. While there is a wealth of literature relating to data analytics, internal audit and human resources individually, the same cannot be said about the literature relating the 3 concepts as a whole. These research gaps are a limitation as there are not many references available to address the topic under study.

In addition, another limitation of the study is the difficulty of proposing a practical model based on theoretical concepts. Therefore, further research is needed in this respect in future lines of work.

### 4.2. Future lines of research

In line with the above limitations, further research is considered necessary in two main areas. On the one hand, more work is needed on improving traditional internal audit processes through new information technologies, specifically data analytics. On the other hand, the theoretical nature of the proposed model represents a great opportunity to be verified through its application in a real case study.

Both lines of research would complement the current literature and provide greater knowledge to a sector in growing development such as internal auditing.

## Data availability statement

The original contributions presented in the study are included in the article/supplementary material, further inquiries can be directed to the corresponding authors.

## Author contributions

All authors listed have made a substantial, direct, and intellectual contribution to the work and approved it for publication.

## Conflict of interest

The authors declare that the research was conducted in the absence of any commercial or financial relationships that could be construed as a potential conflict of interest.

## Publisher’s note

All claims expressed in this article are solely those of the authors and do not necessarily represent those of their affiliated organizations, or those of the publisher, the editors and the reviewers. Any product that may be evaluated in this article, or claim that may be made by its manufacturer, is not guaranteed or endorsed by the publisher.

## Abbreviations

IAIE: Instituto de Auditores Internos de España
IIA: Institute of Internal Auditors
FECYT: Fundación Española para la Ciencia y la Tecnología
WoS: Web of Science
SOX: Sarbanes-Oxley
DEA: Director de Auditoría Interna
ERP: Enterprise Resource Planning
